# Complement and kidney diseases: unlocking the opportunity of targeted treatments for glomerular diseases, including IgA nephropathy

**DOI:** 10.1007/s00467-026-07166-0

**Published:** 2026-02-20

**Authors:** Joshua Wade, David C. Thomas, Matthew C. Pickering, Nicholas R. Medjeral-Thomas

**Affiliations:** 1 Cambridge Institute of Therapeutic Immunology and Infectious Disease, University of Cambridge, Cambridge, UK; 2 Department of Immunology and Inflammation, Faculty of Medicine, Imperial College London, London, UK; 3 Respiratory, Immunology and Inflammation Research Unit, Medicine Research Centre, GSK, Stevenage, UK

**Keywords:** Complement, Kidney diseases, IgA nephropathy

## Abstract

The imminent availability of multiple therapeutic complement inhibitors, which target different complement pathway components, could revolutionise treatment for a broad range of kidney diseases. However, the complexity of complement activity within and between kidney diseases, for which IgA nephropathy is an illustrative example, and the possible adverse effects of complement inhibition mean robust patient selection and stratification to appropriately targeted inhibitors will be needed to maximise this therapeutic opportunity. Despite promising candidates, novel biomarkers that stratify patients to targeted complement inhibition have not yet been validated for clinical practice.

## Introduction

We are in an era of complement therapeutic development. Based on improved understanding of how complement dysregulation contributes to kidney disease pathogenesis, molecules that target almost every facet of the complement system are in clinical and pre-clinical trials. The high prevalence of glomerular complement deposition in a range of kidney diseases demonstrates the importance of complement activation to kidney disease pathogenesis. Our appreciation that complement is more than a marker of complement-fixing immune complexes in kidney diseases [1] was triggered by the demonstration that some progressive kidney diseases are caused by dysregulated, pathogenic complement activity, in the absence of antibody, immunoglobulin, or immune complex [2]. These insights, particularly in the pathogenesis of complement-mediated thrombotic microangiopathy (cTMA, also referred to as atypical haemolytic uraemic syndrome, aHUS) and C3 glomerulopathy (C3G), triggered a treatment paradigm shift and sparked research in the larger range of kidney diseases with detectable complement activity.

This review will focus on the development of therapeutic complement inhibitors for glomerular diseases. First, we will describe the current understanding of the complement system. Next, we will examine evidence of pathological complement activation across a spectrum of glomerular diseases, incorporating evidence from recent clinical trials. We will describe the development and demonstration of the therapeutic benefit of complement inhibitors in cTMA and C3G. However, we will focus on glomerular diseases in which there is evidence of multiple, complex complement mechanisms and pathway activity, in particular IgA nephropathy (IgAN), but also lupus nephritis (LN) and ANCA-associated vasculitis (AAV). For these glomerular diseases, targeted complement inhibition, for example with lectin complement pathway inhibitors, could deliver effective and relatively safe therapeutic benefit. However, realisation of this therapeutic potential is dependent on understanding the role of and identifying activity of specific complement pathway arms and mechanisms . This leads to a discussion of the potential role for biomarkers to facilitate precision medicine selection of the most appropriate complement inhibitor for kidney disease sub-cohorts and individual patients.

## Overview of complement

**Fig. 1:**
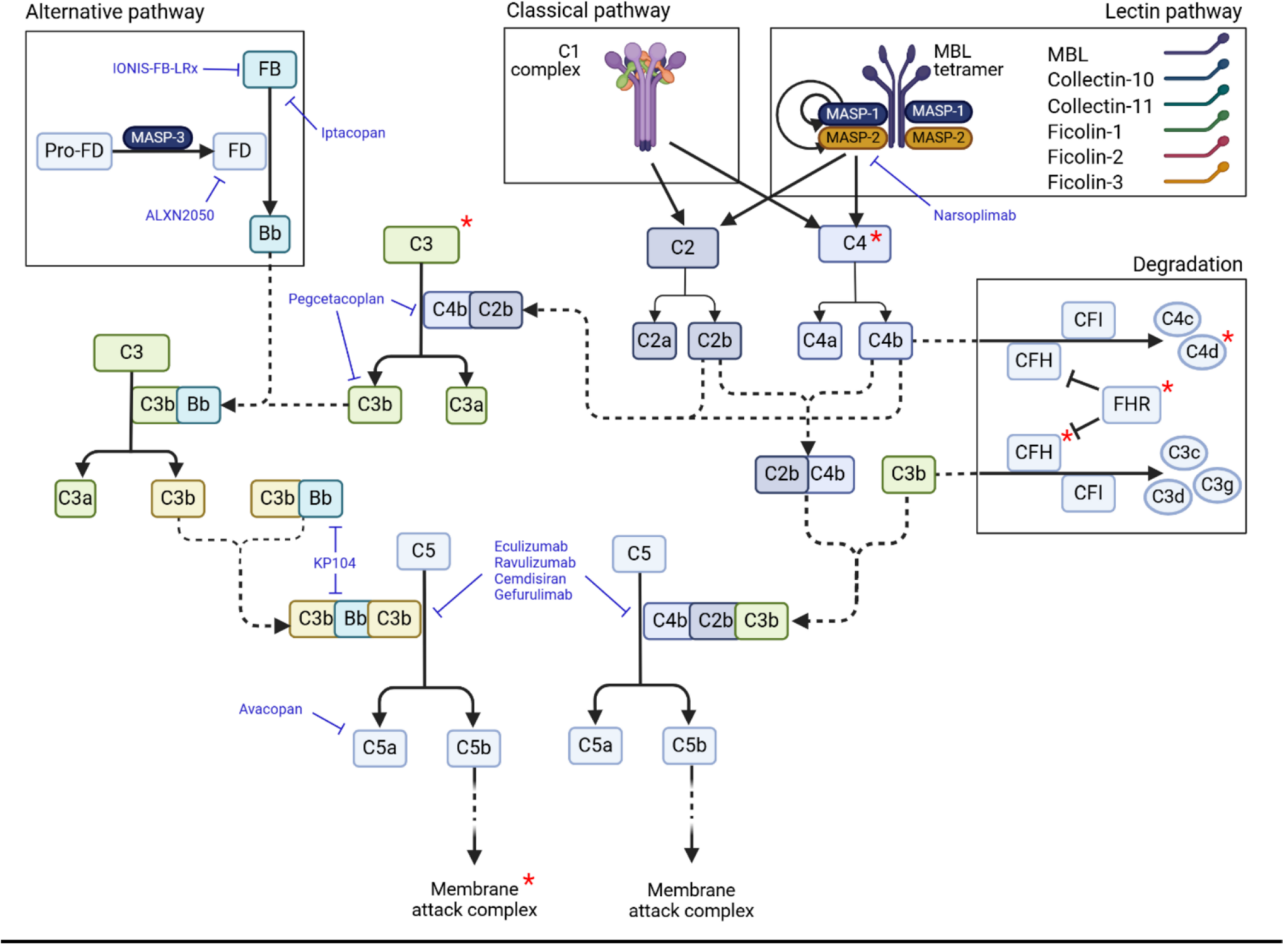
The complement pathway, drugs acting upon it, and potential biomarkers. *Potential biomarker components. Drugs to complement pathway targets are shown in blue. Created in BioRender [3]

The complement system is a proteolytic cascade that encompasses three activation pathways, the classical, alternative, and lectin pathways, which converge at the level of C3 activation and activate the terminal pathway (Fig. 1).

## Classical pathway

The classical pathway forms a link between adaptive, antibody-mediated immunity and innate effector mechanisms. It contains multiple proteins as follows: C1 is a complex formed of C1q, two molecules of C1r, and two molecules of C1s, which are physiologically all pre-bound [4] and also referred to as C1qC1r_2_C1s_2_. C1q recognises the Fc portion of IgG and IgM antibodies complexed with antigen and triggers a conformational change in the complex. This activates the enzymes C1r and C1s. C1q can also bind to apoptotic material and appears to be important in the clearance of molecular debris [5]. Active C1s converts C4 (a substrate that covalently attaches to surfaces) into C4a and C4b; the enzyme C2 then further binds to C4b and is cleaved by C1s into C2a and C2b. C4b and C2b associate to form the C3 convertase C4bC2b. (Confusingly, C2b used to be referred to as C2a, so this complex is also often referred to as C4b2a.) C4b associates with cell surface glycoproteins or is rapidly hydrolysed via an exposed thioester bond. C4b is cleaved by Factor I, first into inactive iC4b and then into C4c, which is soluble, and C4d, which remains membrane-bound [6]. The duration that C4d remains detectable in tissue after activation is not known. Further negative regulation is provided by C1 inhibitor (C1INH), which dissociates C1r and C1s from C1q, and by decay-accelerating factor (CD55), a membrane protein which accelerates the dissociation of C4b and C2b.

## Lectin pathway

The lectin pathway is in many ways analogous to the classical pathway: many components are structurally similar, and it leads to the formation of the C4bC2b convertase. However, lectin pathway activity has different triggers than the classical pathway. Pattern recognition molecules (PRMs) like collectins and ficolins directly recognise and interact with pathogen carbohydrate molecules, and activation is initiated by PRM-bound serine proteases MASP-1 and MASP-2. There are currently six identified lectin PRMs: mannose binding lectin (MBL); two collectins, collectin-10 (collectin liver-1, CL-L1) and collectin-11 (collectin kidney-1, CL-K1); and three ficolins, ficolin-1 (M-ficolin), −2 (L-ficolin), and −3 (H-ficolin) [7]. These form homo- or heterodimers and cause autoactivation of MASP-1 which in turn cleaves and activates MASP-2 and C2. MASP-2 also has some auto-activation ability and appears to be sufficient for lectin pathway activation alone [8]—it cleaves both C2 and C4, leading to the C4bC2b convertase as in the classical pathway. MASP-2 is the only part of the lectin pathway capable of cleaving C4 [9].

The *MASP1* gene encodes MASP-1, MASP-3, and MAp44 (MAP-1) via alternative splicing [10] and the *MASP2* gene encodes both MASP-2 and MAp19(MAP-2) [11]. MAp19 and MAp44 lack the C-terminal proteases of the MASP proteins and are thought to inhibit lectin pathway activation by competing with MASP-1 and MASP-2 for binding sites on PRM complexes. MASP-3 also binds to PRM complexes [12] but its only known function relates to the alternative pathway and will be discussed later. Further inhibitory regulation of the lectin pathway is thought to be mediated via C1INH, antithrombin, alpha-2-macroglobulin, and inter-alpha-trypsin inhibitor heavy chain [7]. Lastly, there is some in vitro evidence of antibody-mediated activation of the lectin pathway [13] which will be discussed later in more detail with reference to disease states.

## Alternative pathway

The alternative pathway involves constituently active ‘tickover’ and provides rapid amplification of complement activity, regardless of the triggering pathway branch. Importantly, alternative pathway activation does not require immune complexes or pathogen molecules but can be initiated by the spontaneous hydrolysis of an internal thioester bond within intact C3. The activated C3 molecule from alternative pathway tick-over, which involves hydrolysis of the thioester bond, is termed C3H_2_O. Unlike intact C3, C3H_2_O can bind to Factor B (FB) to form a C3 pro-convertase. The bound FB is then cleaved by Factor D (FD) to generate an active C3 convertase (C3H_2_OBb). This convertase cleaves intact C3 into C3b and C3a. Like C3H_2_O, C3b can bind to FB and generate a C3 convertase (C3bBb). C3b generated via any of the pathways can result in a rapid increase in C3b by interacting with FB and FD (the C3b amplification loop) [14].

Notably, MASP-3 is usually active in plasma and cleaves pro-Factor D to the active and more abundant form, FD [15]. C3a is an anaphylatoxin which binds to C3aR on a variety of immune cells. Properdin is a positive regulator which stabilises the C3bBb complex, prevents dissociation, and allows further C3b to associate, forming C3bBbC3b, a C5 convertase [14].

The key negative regulators of the alternative pathway and the C3b amplification loop are Factors I and H. Factor H (FH) dissociates C3 convertases and prevents their assembly. With cofactors, FI proteolytically cleaves C3b to iC3b and then C3d. While both iC3b and C3d have biological functions through receptor engagement, neither can interact with FB, so the proteolysis of C3b prevents further C3b generation. Other regulators include complement receptor 1 which binds C3b and C4b and acts as a cofactor for the FI-mediated degradation of both proteins. FH is part of a protein family which includes Factor H–related (FHR) proteins. The FHR proteins, in contrast to FH, appear to promote C3b activation [16]. Membrane cofactor protein (MCP, CD46) is a membrane-bound cofactor for Factor I–mediated proteolysis of C3b and C4b [14].

## Terminal pathway

The binding of an additional molecule of C3b to C3 convertases results in complexes that can cleave complement C5. This results in the production of C5b and C5a and is the first step in the terminal pathway. C5a is an anaphylatoxin which binds the C5a receptor (C5aR) found on a wide range of immune cells, while C5b associates with C6, C7, C8, and multiple C9 molecules to form the membrane attack complex (MAC), which has direct cytotoxic activity especially important in defence against encapsulated bacteria. The key negative regulator in this pathway is CD59, a membrane-bound protein that prevents the assembly of the MAC [17].

## Complement and kidney disease

Recent and ongoing research into the complement system implicates complement dysregulation in the pathogenesis of numerous diseases, which have in turn fuelled interest in complement as a therapeutic target. The glomerular basement membrane (GBM) appears to be particularly susceptible to complement dysregulation and subsequent pathology. Glomerular filtration and endothelial cell fenestration may lead to a high concentration of complement proteins in the GBM vicinity, which, coupled with a lack of cell membrane–based regulators on the GBM, could explain this susceptibility [18].

To illustrate the spectrum of diseases affected by complement, we will describe the evidence for complement system activity in the pathogenesis of a non-exhaustive selection of glomerular diseases. We will start with rare diseases for which pivotal research has demonstrated a causative role, cTMA, and C3G. We will next focus on more common glomerular diseases with evidence of dysregulated complement activity in multiple complement pathway arms. These include IgAN, LN, and anti-neutrophil cytoplasmic antibody (ANCA)-associated vasculitis (AAV). In an era of multiple therapeutic inhibitors to different pathway components, these glomerular diseases present an opportunity for targeted, individualised, effective treatment. However, there is also a risk that mistargeted use could result in non-positive clinical study data, limiting the use and availability of potentially efficacious treatments for important kidney diseases.

## Complement-mediated thrombotic microangiopathy

Complement-mediated TMA presents clinically as a triad of microangiopathic haemolytic anaemia, thrombocytopenia, and acute kidney injury. It is a rare disease with an incidence of 0.41 cases per million per year in the UK [19] but has proved instructive in showing how an understanding of complement dysregulation can lead to treatment.

Genetic mutations and variants in complement alternative pathway proteins associate with cTMA. This observation led to research that demonstrated dysregulated C3 convertase activity leads to excessive alternative and terminal pathway activity, endothelial cell damage, platelet recruitment and activation, and kidney microangiopathic thrombosis. This uncontrolled C3 activation occurs at the cell surface, primarily due to disruption of regulatory proteins. Variants in genes of the regulator FH account for approximately 25% of genetic cases, and variants in FI account for 5–10% and CD46 for a further 10% of cases [20]. Activating mutations of C3 and FB are also seen. Interestingly, the penetrance of the best characterised variants is only 50% [21] suggesting that alone they are not sufficient to cause disease, and cTMA is a complex oligogenic disease. Autoantibodies that interfere with complement regulatory proteins have also been identified, and there is a link between a deletion spanning the genes encoding FHR1 and FHR3 and anti-FH autoantibodies [22].

Further evidence for complement-related pathogenesis comes from transgenic mouse models with disruption of FH [23] or gain-of-function C3 mutation leading to cTMA. In the latter, C5 cleavage inhibition or C5 knock out was shown to prevent disease [24]. In phase 2 trials, eculizumab, a C5 cleavage inhibitor, shows a substantial improvement in eGFR [25] and a persistent benefit in kidney survival at 5 years in retrospective analysis [19]. Eculizumab has been approved by regulators for this indication and is being actively investigated in other conditions. Ravulizumab is a newer C5 inhibitor which is also licenced for cTMA. It has a longer half-life and so requires less frequent infusions [26].

## C3 glomerulopathy

C3G is caused by alternative pathway dysregulation [27] and is considered when kidney biopsy shows complement C3 staining at least 2 orders of magnitude greater compared to any other immune reactant [28]. Two major subtypes are dense deposit disease and C3 glomerulonephritis. It is rare with an incidence of 1–3 per million [29].

The finding of C3 deposition (C3b, iC3b, C3c, and C3dg) without significant immunoglobulin on kidney biopsy suggests alternative pathway activation. A low plasma C3 level with normal C4 levels may also be seen. Known causes of C3G include C3 nephritic factors (50–80%), gain-of-function mutations in C3 or FB (very rare), and abnormal FHR proteins (very rare) [29]. C3 nephritic factors are antibodies which stabilise the C3 convertase and prevent its inhibition by FH. Whether they are directly pathogenic remains unclear. It is important to note that in older individuals with C3G, there is an association with paraproteins, and in many cases, no cause is identified.

Immune complex membranoproliferative glomerulonephritis (IC-MPGN) shares histological features with C3G but exhibits IgG deposition on kidney biopsy which may be accompanied by C3. IC-MPGN may be primary or secondary to other disease including infection, autoimmune conditions, and monoclonal gammopathy. Nephritic factors also occur in IC-MPGN, and there is significant overlap with C3G with instances of biopsies from one patient being consistent with each condition at different times [30].

Consistent with a key role of complement C3 activation in the pathogenesis of C3G, pegcetacoplan, a C3 and C3b inhibitor, and iptacopan, an FB inhibitor, showed a reduction in proteinuria in phase III trials [31, 32]. Iptacopan and pegcetacoplan have been approved by the US Food and Drug Administration (FDA) [33]. C5 blockade appears to be insufficient to prevent kidney failure in C3G and IC-MPGN although the benefit may be limited to ameliorating kidney injury in patients with inflammatory changes [34–36]. ACCOLADE was a Phase II trial of avacopan (a C5aR1 antagonist) over placebo which used a histological measure of C3 activity at 26 weeks as a primary end point [37]. Despite a reduction in this measure with avacopan, the disease still progressed, and further trials were halted.

## IgA nephropathy

IgA nephropathy (IgAN) is the most common glomerulonephritis with an estimated incidence of 2.5 cases per 100,000 per year [38]. It is slowly but relentlessly progressive with a median time from diagnosis to kidney failure of 10.7 years and a median age at kidney failure of 55 years in the UK according to National Registry of Rare Kidney Diseases data [39].

The pathophysiology of IgAN is described as a ‘4-hit’ process involving galactose deficient IgA1 (gd-IgA1); IgG and IgA autoantibodies; formation of polymeric IgA1 complexes; and deposition of immune complexes, with complement activation in the glomerulus [40]. Histologically, glomerular deposition of C3 and other complement products is seen alongside polymeric gd-IgA1 with some cases also exhibiting IgG and IgM. While IgG may be under-detected (it has been extracted via enzyme-linked immunosorbent assay [41] from biopsies that appeared negative on immunofluorescence), there is increasing evidence of a direct role of IgA1 in complement activation and tissue inflammation.

Evidence for complement involvement in IgAN pathophysiology is based on histological review of kidney biopsies coupled with genome wide association studies (GWAS), measurement of plasma and urine complement components and in vitro studies. Evidence of multiple complement pathway arm activity has been identified in IgAN, making IgAN an ideal exemplar disease to consider targeted treatment opportunities and challenges for complement inhibitors.

The alternative pathway appears most activated in IgAN. For example, in a study of 60 IgAN biopsies in an Asian cohort, 87% had Factor Bb deposition and 75% had properdin deposition [42]. GWAS showed that a copy number polymorphism resulting in deletion of the *CFHR1* and *CFHR3* genes conferred protection from IgAN [43–46]. Reduced plasma levels of C3 have been correlated with IgAN progression [47]. Lastly, there is in vitro evidence for direct activation of the alternate pathway via polymeric IgA [48].

Alternative pathway activation is thought to occur in the majority of IgAN cases and is appreciable from routine clinical diagnostic immunostaining of kidney biopsies. Lectin pathway activation is not routinely assessed in clinical samples; however, multiple research studies have identified evidence of lectin pathway activity in a proportion of IgAN patients (Table 1). If pathogenic lectin pathway activity can be identified, its suppression is a potentially safe approach to therapeutic complement inhibition given the reduced infection liability predicted with lectin pathway suppression, compared to alternative and terminal pathway suppression, in adults. The identification in IgAN of lectin pathway components, including MBL, collectin-11, ficolin, MASPs, and C4d (Table 1) indicate opportunity for pathogenic pathway biomarker development. C4d is a product of both the classical and lectin pathway, but not the alternative pathway, whereas C1q is integral to the classical pathway, and C4d deposition in the absence of C1q is therefore likely the result of lectin pathway activation [49]. That said, the duration of C4d and C1q tissue deposition is not known, and a longer duration of C4d and C1q could lead to a false assumption of absent classical pathway activity. MBL staining is positive in 17–34% [40] of IgAN biopsies and tends to be found with other lectin pathway components including MASP-2. Discontinuity between MBL and C4d staining in IgAN biopsies can be explained by the contribution of other PRMs: staining for MBL, collectin-11, and L-ficolin gives high agreement with lectin pathway components and C4d staining [42].

**Table 1:** Published IgA nephropathy kidney biopsy evidence of lectin pathway activation

Study	Cases	Lectin pathway component	Percentage positive	Notes
Matsuda [50]	42	MBL	16.7%	
Endo [51]	45	MBL	24.4%	
Lhotta [52]	11	MBL	27%	Focal and segmental pattern of staining interpreted as non-specific entrapment
Hisano [53]	36	MBL MASP-1	52.8% 52.8%	
Roos [54]	60	MBL	25%	All samples positive for MBL were also positive for L-ficolin, MASP-1/3, C4d and C4-BP
Liu [55]	131	MBL	34%	
Wei [42]	60	MBL L-ficolin collectin-11 MASP2 MASP1/3 C4d	40% 55% 37% 58% 48% 67%	63% positive for at least 1 lectin pathway component 12% were positive for at least 1 lectin pathway component and C1q. All of these were also Bb positive
Sahin [56]	33	C4d	33%	
Espinosa [57]	59	C4d	32%	All patients C1q negative
Maeng [58]	23	C4d	57%	
Espinosa [59]	283	C4d	38.5%	All patients C1q negative
Faria [60]	74	C4d	34%	
Heybeli [61]	37	C4d	43%	
Wągrowska-Danilewicz [62]	43	C4d	25.6%	C1q not reported
Fabiano [63]	47	C4d	21%	
Seggara [64]	190	C4d	20%	All patients C1q negative
Baek [65]	56	C4d	55%	
Sato [66]	25	C4d	56%	
Nam [67]	380	C4d	19%	
Yang [68]	642	C4d	6.4%	Of which 61% C1q positive (*N* = 25/41 of C4d had C1q)

Although IgAN GWAS have not identified evidence of lectin pathway activation, an analysis of MBL2 polymorphisms in 1007 IgAN patients identified an allele associated with an increased risk of kidney failure [69]. Studies of plasma levels of lectin pathway components have proved difficult to interpret with both high and low levels of MBL associated with severity [70]. Finally, there is evidence for polymeric IgA activating the lectin pathway in vitro [13, 54] in a rat model [71] via MBL and via collectin-11 in vitro [42]. The interaction between MBL and IgA involves the lectin domain of MBL and likely involves a carbohydrate ligand.

Degalactosylation of IgA appears to be important in revealing this binding site [72] and pooled IgA1 immune complexes from patients with IgAN appear to increase the release of collectin-11 and inflammatory factors IL-6 and MCP-1 from mesangial cells in vitro when compared with IgA1 immune complexes from healthy controls [42].

Based on immunohistology detection of C1q in a small proportion of IgAN biopsies only, the classical pathway is not thought to contribute significantly to IgAN pathogenesis. Evidence of the terminal pathway of complement is found in the majority of biopsy samples [42] and correlates with disease severity [73].

It cannot be determined with certainty whether activation of different complement pathways represents the same disease pathophysiology sampled at different timepoints or different subcategories of disease. Most studies do not report estimated duration from onset of disease symptoms, which is difficult to determine due to the often insidious onset of IgAN. Of the few repeat biopsy reports, C4d deposition (and so presumed lectin pathway activation) does not appear to resolve but can be detected in previously C4d negative patients [64]. Although C4d is covalently bound in tissue and relatively long-lived on an extracellular matrix, it is short-lived on nucleated cells and has been shown to fluctuate in protocol transplant biopsies (albeit in the peritubular capillary) [74], so it is plausible to extrapolate that deposition represents ongoing complement activation.

Correlations between complement pathway activation and demographic characteristics have been inconclusive; a non-statistically significant trend towards higher C4d deposition in the Asian population [75] is the most durable association identified. Nevertheless, which specific complement pathways are activated appears to influence clinical severity and outcomes. C4d positivity consistently correlates with worse disease [76], as evidenced by proteinuria and histological severity at the time of biopsy, and an increased risk of severe kidney disease. A meta-analysis of C4d deposition calculated a risk ratio (RR) of 3.37 for kidney failure [75]. Glomerular FHR5 deposition has also been shown to correlate with progressive disease [76]. In summary, evidence of lectin and alternative pathway activation associates with more aggressive disease, making these appealing therapeutic targets.

## IgAN clinical trials

### Approved treatments

Aside from supportive measures that target the non-disease specific consequences of nephron loss and chronic kidney disease (CKD), there has been a paucity of treatment options to address the significant morbidity of IgAN [77]. Increasing understanding of the complement pathway and its importance in IgAN has led to interest in the use of therapeutic complement inhibitors for IgAN. In reflection of the complexity of complement activity in IgAN, therapeutic inhibitors of multiple different pathway arms are in development (Table 2). The tractability of IgAN drug development improved with the establishment of proteinuria reduction as a surrogate endpoint for progression to kidney failure and its acceptance for accelerated regulatory approval [78]. Consequently, two new drugs, including a complement inhibitor, have been approved for IgAN treatment: targeted release formulation budesonide (kinpeygo or tarpeyo) [79] and a FB inhibitor (iptacopan) [33].

**Table 2:** Examples of IgA Nephropathy clinical trials of novel therapeutic complement inhibitors that demonstrate targeting of multiple, different complement pathway arms

Drug	Complement pathway	Target	Trials
Ravulizumab	Terminal	C5	Phase 2. NCT04564339 (IgAN and LN) [80]
Cemdisiran	Terminal	C5	Phase 2. NCT03841448 [81]
Gefurulimab	Terminal	C5	Phase 1B. NCT05314231 (proteinuric disease including IgAN) [82]
Avacopan	Terminal	C5aR	Phase 2 NCT02384317 [83]
Iptacopan	Alternative	Factor B	Phase 3 NCT04578834 [84]
IONIS-FB-LRx	Alternative	Factor B	Phase 3 NCT05797610 [85]
ALXN2050	Alternative	Factor D	Phase 2 NCT05097989 (IgAN and LN) [86]
KP104	Alternative and terminal	C3bBb, C5	Phase 2 NCT05517980 (IgAN and C3G) [87]
Pegcetacoplan	Alternative	C3	Phase 2 NCT03453619 (C3G, IgAN, LN and PMN) [88]
Narsoplimab	Lectin	MASP-2	Phase 3 NCT03608033 [89]

### Alternative pathway inhibitors

Iptacopan was approved for IgAN by the FDA based on interim analysis of the phase 3 APPLAUSE-IGAN study. APPLAUSE-IGAN recruited patients at high risk of progression as indicated by a high level of proteinuria (urinary protein creatinine ratio ≥ 1 g/g on 24 h collection) and has so far shown a 38.3% reduction in proteinuria at 9 months while being well tolerated [84]. This is consistent with the premise that the majority of IgAN involves dysregulated alternative pathway activity.

Other clinical trials targeting the alternative pathway are underway including IONIS-FB-LRx, an antisense oligonucleotide to FB synthesis; ALXN2050, a small molecule FD inhibitor; KP104, FH fused to an anti-C5 antibody and so targeting the C3bBb convertase and terminal pathway (Table 2). A phase II trial of pegcetacoplan, which binds and inhibits C3 and C3b, in a variety of kidney diseases showed no significant change in proteinuria for IgAN [88]. Several other C3 inhibitors are actively in development including AMY101 and ADX-097 (an anti-C3d antibody combined with a fragment of FH), but these have not yet been trialled in IgAN patients.

In addition to delivering efficacious treatment options, data from alternative pathway inhibitor trials could reveal the pathogenic relevance of alternative and lectin pathway activity in IgAN. Based on immunohistology, a subset of IgAN patients are expected to have lectin pathway activation, either alone or in combination with alternative pathway activation. Since C4d deposition is correlated with higher proteinuria, lectin pathway activation might be enriched in trial cohorts, and it will be interesting to see if there is a subgroup of IgAN patients with disease that is resistant to alternative pathway inhibition.

### Lectin pathway inhibitors

MASP-2 has been identified as a drug target because it is the only lectin pathway component which can cleave C4 and is therefore thought to be essential for lectin pathway activity [8]. In addition, MASP-2 can autoactivate (but this has only been shown at supraphysiological concentrations) and there is some evidence for C4/C2 bypass with direct activation of C3, although the mechanism is unknown [90]. MASP-2 has also been shown to cleave prothrombin [91] and thus may increase clot formation and potentially promote thrombotic microangiopathy, a pathology characteristic common to severe glomerular diseases. Encouragingly, MASP-2-deficient mice have no increased susceptibility to *Pseudomonas aeruginosa* [92]. Patients homologous for a *MASP-2* gene mutation associated with no lectin pathway activity do not appear to have an increased propensity to primary immunodeficiency despite initial reports to the contrary [93]. Taken together, this suggests MASP-2 inhibition may not significantly increase infection risk.

Narsoplimab is an anti-MASP-2 antibody which had a favourable phase II trial [94] in IgAN, but the phase III trial, ARTEMIS-IGAN [89], was stopped early due to a lack of divergence between the treatment and placebo groups [95]. Like APPLAUSE-IGAN, ARTEMIS-IGAN recruited patients with high rates of proteinuria who should be at increased risk of disease progression and used reduction in proteinuria as an outcome measure. Despite enriching for high-risk patients, it may be that a minority of recruited patients had lectin pathway activation (Table 1) and that any potential treatment effect was therefore diluted. Also, a less frequent dosing regimen was used in ARTEMIS-IGAN than in the phase 2 narsoplimab study, and we do not know whether lack of pharmacology engagement contributed to negative phase 3 data. An inability to accurately measure the degree of complement blockade is an important challenge in clinical trial design.

### Terminal pathway inhibitors

Targeting the terminal pathway is an attractive therapeutic approach because it is the common endpoint of all complement pathways and its effector functions can cause tissue injury. Initial case reports of eculizumab use in severe IgAN showed some signs of treatment response, but the results were not definitive [96–98]. Trials of other terminal pathway inhibitors ravulizumab, cemdisiran, and gefurulimab, and the C5aR blocker avacopan, have shown some efficacy [80–83]. However, terminal pathway inhibitors will not impact more proximal effector molecules (such as C3a and C3b), which have independent pathologic, pro-inflammatory effects. Increased susceptibility to, and severity of, infections with encapsulated bacteria in the context of C5 inhibition also remains a concern.

Complement activation inhibition is not expected to prevent gd-IgA production or glomerular IgA deposition. Therefore, complement inhibition aims to reduce glomerular inflammation and complement deposition, despite persistent IgA deposition. The extent to which IgA deposition alone contributes directly to inflammation and kidney damage is not known.

## SLE and lupus nephritis

Systemic lupus erythematosus (SLE) is a complex, life-long, multisystem disease where patients often suffer from a high disease and treatment burden. Thirty to sixty percent of patients with SLE develop LN of which 30% develop kidney failure within 15 years [99]. The pathophysiology is incompletely understood. An interplay of genetic and environmental factors leads to a breakdown of immune tolerance to nucleic antigens resulting in the generation of multiple anti-nuclear autoantibodies of varying pathogenicity, coupled with a type I interferon response [100]. Many factors have been implicated in this loss of tolerance, including problems with clearance of apoptotic cell material and immune complexes, and there is evidence that complement is involved in this process [101]. In keeping with this, C3 and C4 consumption is seen during flares of disease.

The classical pathway is strongly implicated in SLE pathogenesis, being directly activated by immune complexes. Genetic deficiencies of the C1q complex, C2, and C4 are associated with SLE [102] and biopsies in LN classically show a ‘full house’ of deposition with C1q, which indicates classical pathway activation. The role of complement in lupus pathogenesis appears to be double-edged; while deficiencies in complement components increase the risk of disease, likely via deficiencies in C1q opsonisation of apoptotic debris and immune complexes [103], conversely, active disease is characterised by increased complement activity and deposition.

This pathogenic paradox also appears to apply to the lectin pathway. MASP-1 has been reported to be both raised and reduced in SLE compared to healthy controls, while MASP-2 appears more reliably raised in SLE and to correlate with increased activity [104–106]. Mirroring the classical pathway, low MBL levels appear to be associated with an increased risk of SLE and LN [107–109] and specific risk alleles have been characterised. It has been posited that the lectin pathway also has a role in clearing apoptotic cells and cell debris such that deficient complement leads to more auto-antibody production [110]. Established auto-antibodies can then cause damage via complement activation largely via the classical pathway [111] and possibly the lectin pathway. A small study of 11 LN biopsies which looked for PRM of the lectin pathway found MBL in 9 and L-ficolin in 7 of the biopsies along with C1q [112].

The alternative pathway is important in mouse models of lupus. Lupus-prone mice, which are deficient in the alternative pathway regulator, Factor H, display more severe disease [113]. Inhibiting the alternative pathway by targeting Factor D or the terminal pathway via C5 appears to protect against kidney disease in lupus-prone mice [114, 115].

## Lupus clinical trials

As in IgAN, a number of complement inhibitors are being tested in SLE and LN clinical trials. Many of the IgAN trials already discussed are also recruiting patients with LN, including ALXN2050 (a FD inhibitor), pegcetacoplan (a C3 inhibitor), and ravulizumab (a C5 inhibitor) [80, 86, 88]. There is a case report of successful eculizumab use [116]. Iptacopan, having received accelerated approval for IgAN, is in a phase II trial for LN (NCT05268289) [117]. KP104 which targets the alternative pathway is also being trialled in both diseases [87, 118]. The classical pathway is targeted by ANX009 which inhibits C1q-substrate interactions. A phase 1b trial (NCT05780515) has completed [119].

It is reasonable to hypothesise that SLE encapsulates a spectrum of disease and that the pathogenic contribution of each complement pathway varies between patients and perhaps over time. Once again, detection of a treatment effect may depend on the composition of patients in the trial and whether they have dysfunction of the specific complement component being targeted.

## ANCA-associated vasculitis

One of the diagnostic histological features of AAV is the ‘pauci-immune’ lack of deposition of immunological products and, consequently, AAV is not traditionally thought of as a complement-mediated disease. However, there is a growing appreciation of the role of complement in AAV driven by greater understanding of pathogenesis and the success of complement-targeting treatments.

In a mouse model, injection of myeloperoxidase (MPO)-ANCA causes a necrotising crescentic glomerulonephritis like that seen in humans [120]. Multiple knock outs in this model have shown that alternative pathway complement activation drives disease via the terminal pathway, and particularly C5a. Knock outs of FB, C5, and C5aR are all protective against disease while the knockout of C4 does not prevent pathology [121]. Anti-C5 antibody pretreatment is also effective in attenuating disease [122]. Studies of sera from patients with AAV show increased levels of C4a, C5a, and FB in active disease compared to remission, consistent with this hypothesis [123].

A strong role for C5a is reinforced by the success of the anti-C5aR1 inhibitor avacopan. ADVOCATE was a phase III trial comparing avacopan with prednisolone in remission maintenance, which showed superiority at 52 weeks and is therefore consistent with complement playing an important role in AAV [124]. The mechanistic rationale for the therapeutic effect is that C5a has strong chemoattractant effects and further stimulates recruited neutrophils, causing ANCA targets (MPO and Proteinase 3) to move to the cell membrane where they can be recognised by autoantibodies.

A wider role for complement in AAV pathogenesis is now being re-considered. Despite the label of pauci-immune, 25–54% of affected biopsies have been reported to contain complement deposits [125]. There is not complete agreement across studies, but C3 degradation products are most reported, albeit at low levels, while C4 is usually, but not exclusively, absent. This has also been demonstrated by microdissection and mass spectrometry proteomic analysis of kidney tissue [126]. Factor Bb has been reported in AAV biopsies and appears to correlate with histological markers of severity [127]. It has been hypothesised that rapid degradation in the early clinical phase of disease accounts for the rarity of Bb detection.

## Precision medicine opportunity: complement inhibition for kidney diseases

Therapeutic complement inhibitors may only be efficacious for the sub-cohort of each disease with ongoing activity of the relevant, targeted complement component. This would make treatment success dependent on accurately targeting complement inhibitors to the appropriate pathogenic complement mechanism, regardless of the morphology-based kidney diagnosis. Furthermore, markers of successful suppression of complement activity and clinical progression could direct safe treatment cessation. The next section will discuss biomarkers that could detect disease sub-cohorts driven by specific, targetable complement pathway mechanisms.

## Biomarkers

Non-invasive biomarkers of complement activity would allow serial monitoring of disease activity and response to treatment. However, plasma complement factor levels present an incomplete picture of systemic complement activation and may bear no relation to activation in tissue. Reduced levels may reflect increased consumption in active disease or underlying constituently low levels. Similarly, changes in the concentration of urinary biomarkers may be affected by consumption, deposition, and sequestration in the kidney and changes in passage across the glomerular basement membrane and characteristics of the tubular filtrate. Plasma and urine biomarkers often give incomplete or contradictory results across different studies, reflecting this complexity. Trials of inhibitors are an opportunity to examine these dynamics. In the phase II trial of iptacopan in C3G, one cohort used a primary endpoint based on C3 deposition on kidney biopsy while collecting secondary endpoint data on proteinuria, eGFR, and serum C3 concentrations among others. Plasma C3 levels were observed to rise and C3 deposition to fall [128]. A similar approach, incorporated into the trials of other complement inhibitors, could provide insights into complement-driven pathology. Potential complement-related biomarkers in serum, urine, and on kidney biopsies across the range of diseases discussed above are shown in Table 3.

**Table 3:** Complement related biomarkers in kidney disease

Disease	Serum	Urine	Biopsy
C3G	C3 Nephritic factors [129] Factor B breakdown products [130] Alternative pathway activity such as AH50 [131] C5b-9 [130]	C5b-9 [130]	FHR5 [36] C5b-9 [36]
IgAN	IgA1/C3 ratio [132] FHR-1/Factor H ratio [133, 134] Ba [135] MASP-3 [76]	MBL/Creatinine [136] FH/Creatinine [137] C4d/Creatinine [138]	C4d [68] MBL [55] FHR5 [76] C5b-9 [139] (C3, FB)
LN	C4d/C4 [140] MASP-2 [104, 105] C3 and C4 [141] (split products) C1q [142] (Anti-C1q antibodies [143])	C3d/creatinine [144] C5a/creatinine [145]	C1q [146] (C3)
AAV	Bb [147] C3a [147] C5a [147]	Bb [127] C3a [127] C5a [127] C5b-9 [127]	FB [127] Properdin [148]

## Serum and plasma

Commercial assays that measure the activity of specific complement pathways are available such as from WIESLAB®, but they are not widely used in clinical practice. As they measure complement activity in plasma, they may not reflect activity in specific tissues including in the kidney. For example, dysregulated activity at the cell membrane (rather than in plasma) which characterises cTMA may not be detected by these assays.

Other complement factor levels in serum are appealing surrogates of disease activity. IgA1/C3 ratio, FHR-1 levels, and FHR-1/Factor H ratio have all been identified as potentially useful IgAN biomarkers by this approach [149]. The use of ratios of complement breakdown product to complement factor is a logical approach, and C4d/C4 appears promising. In SLE, plasma C4d/C4 was found to be higher than in healthy controls and higher again in lupus nephritis compared to SLE without kidney involvement [140]. Samples were taken at the time of biopsy including post-treatment biopsies. This correlation with validated biopsy features of activity provides good evidence that disease activity via complement activation is being measured. C4d/C4 has been shown to be increased on occasion in IgAN [150] but further evidence to support correlation with disease activity has not been established. Since low C3 and C4 correlate with disease activity in lupus but not the other diseases discussed, complement activation may be more local to the kidney in other diseases, making this measure less useful.

## Urine

Urinary biomarkers present distinct challenges due to the large possible dynamic range, urinary pH, and need to control for both the quantity of urine produced and the effects of other urinary constituents. In IgAN, urine MBL, properdin, FH, and C5b9 associate with other markers of activity [151]. However, a single centre study of urine C4d/creatinine ratio in urine samples paired with kidney biopsy samples failed to show a significant correlation with glomerular C4d deposition. Although there was an association with an increased risk of kidney failure, these data could be confounded by proteinuria correlations. The story is similar in SLE. For instance, urinary C3d/creatinine has been reported as correlating with disease activity and predicting treatment response, but baseline samples were not clearly timed with biopsy samples, and the sample size was small [144], necessitating further validation.

## Kidney biopsy

Staining for complement activation products C3 (which detect C3b, iC3b, and C3c), C1q, and, currently in transplant biopsies, C4d is common practice, but the detection of components unique to the alternative and lectin pathways, such as Factor B and MBL, is not validated for clinical practice. A resource of stored, surplus, formalin-fixed paraffin-embedded samples exists for which amenability to retrospectively performed immunohistochemistry has been established in many large cross-sectional studies.

It may be possible to use biopsies to identify which complement pathway(s) are active and pathogenic, provide prognostic insight, and subsequently direct clinical trial patient selection or randomisation stratification. For example, in IgAN where evidence of specific complement pathway activity is not uniform, information about complement activation from a biopsy could be used to sub-stratify patients to appropriate treatment. A drawback of this approach is that creating a further restricted subpopulation of an already rare disease has implications for clinical trial recruitment.

In summary, there is a strong argument for adding additional complement antigen strains to diagnostic kidney biopsies to enable us to match the disease pathophysiology with the most appropriate complement inhibitor, although the timing of biopsy needs to be considered when using these data for treatment decisions. Blood and/or urine biomarkers that capture changes in complement activity within the kidney would enable us to monitor efficacy without the need for kidney biopsy. Development of these biomarkers is challenging as this requires rigorous verification in independent cohorts, histological correlation (using samples collected at the time of biopsy), and multiple timepoints.

## Conclusion

The role of complement in kidney diseases has been revolutionised by the demonstration that dysregulated complement activity causes and drives kidney injury. Novel therapeutic complement inhibitors have met efficacy endpoints in clinical trials. These include eculizumab/ravulizumab in cTMA, avacopan in AAV, pegcetacoplan in C3G and iptacopan in IgAN and C3G. Molecules that target different components of complement are under active investigation. To maximise future clinical benefit of this important therapeutic opportunity for kidney disease patients, we will need to develop effective stratification tactics and non-invasive complement biomarkers for drug, disease, and patient selection.
